# GT‐Seq Panel Development for Species Identification and Parentage Analysis of Closely Related Hybridising *Scaphirhynchus* Sturgeons

**DOI:** 10.1111/1755-0998.70124

**Published:** 2026-03-27

**Authors:** Junman Huang, Richard Flamio, Nathan R. Campbell, Aaron J. DeLonay, Amy C. Buhman, Edward J. Heist

**Affiliations:** ^1^ School of Biological Sciences Southern Illinois University Carbondale Carbondale Illinois USA; ^2^ Center for Fisheries, Aquaculture, and Aquatic Sciences Southern Illinois University Carbondale Carbondale Illinois USA; ^3^ Department of Science, Dominican University New York Orangeburg New York USA; ^4^ GTseek LLC Twin Falls Idaho USA; ^5^ U.S. Geological Survey Columbia Environmental Research Center Columbia Missouri USA

**Keywords:** conservation genetics, high‐throughput sequencing, introgressive hybridisation, SNP, species complex

## Abstract

Hatchery supplementation is vital for conserving dwindling fish populations. Effective augmentation requires distinguishing hatchery‐origin from wild individuals and accurately identifying species, particularly in systems where closely related species coexist. Genetic monitoring is key to quantifying genetic differences, but conventional markers do not distinguish hybrids, especially backcrosses. Misidentifying hybrids in hatchery programs compromises wild gene pools because hatchery broodstock contributes to numerous offspring being released into the wild. Here, we present a workflow for developing and evaluating the Genotyping‐in‐Thousands by sequencing (GT‐seq) single nucleotide polymorphism (SNP) panel for North American river sturgeons (*Scaphirhynchus* spp.). This panel is designed to detect complex hybrid classes and to determine parent‐offspring relationships. Our species identification panel (S‐loci) contains 155 SNPs selected for high genetic differentiation (F_ST_) between Pallid Sturgeon ( *S. albus* ) and Shovelnose Sturgeon ( *S. platorynchus* ), and the parentage assignment panel (P‐loci) includes 112 SNPs with high heterozygosity within Pallid Sturgeon. Simulation analyses demonstrated that our GT‐seq S‐loci panel reliably classifies pure species, F1, F2 and backcross hybrids, even with up to 70% missing data. The P‐loci panel achieves high‐confidence parentage assignment with ≥ 80% typed loci, with performance influenced by the proportion of sampled parents. Overall, the novel *Scaphirhynchus* GT‐seq panel developed in this study represents a robust and efficient tool for detecting hybridisation, assigning parentage and providing critical information for management decisions in ongoing Pallid Sturgeon conservation.

## 1 Introduction

*Scaphirhynchus*, a wholly riverine genus of sturgeons native to rivers of the central and southern United States, comprises three species that are threatened to varying degrees. Pallid Sturgeon ( *Scaphirhynchus albus* ) inhabits the Missouri and lower Mississippi rivers and was listed as endangered under the U.S. Endangered Species Act in 1990 (USFWS 1990) and as critically endangered by the International Union for Conservation of Nature (IUCN) in 2020 (Jordan and Nelson‐Stastny 2022). Shovelnose Sturgeon ( *S. platorynchus* ) are far more numerous but were listed as threatened under the similarity of appearance clause U.S. Endangered Species Act in 2010 (USFWS 2010) to eliminate harvest of Pallid Sturgeon in Shovelnose Sturgeon fisheries (Phelps et al. 2016). Current threats to Pallid Sturgeon include habitat destruction, overexploitation, disease, inadequate regulation and hybridisation with the much more common Shovelnose Sturgeon (USFWS 2014). Extensive habitat destruction in the Missouri River, including the construction of five major dams and reservoirs in the mid‐20th century, threaten Pallid Sturgeon persistence. Since erection of the dams, Pallid Sturgeon have failed to naturally recruit in the upper Missouri and Yellowstone rivers, most likely due to altered flow, temperature and lack of drift distance for larvae (Braaten et al. 2008; Jordan et al. 2016). Additionally, there has been limited recruitment of Pallid Sturgeon in the lower Missouri basin, where molecular markers (e.g., microsatellites) are used to document that wild‐caught Pallid Sturgeon are both wild and not hybrids (Steffensen et al. 2019). However, these markers lack resolution to reliably distinguish pure species from advanced backcross hybrids (Jordan et al. 2019). Introgressive hybridisation with Shovelnose Sturgeon is much more common in the lower Missouri and Mississippi rivers than in other river basins, further complicating species identification (Allendorf et al. 2001; Schrey et al. 2011). These compounded pressures underscore the need for integrated conservation and management strategies that address both habitat restoration and the genetic integrity of remaining populations (DeLonay et al. 2016).

An integrative management strategy for Pallid Sturgeon conservation has been developed based on analyses of habitat niches, genetic diversity and historical records, resulting in the designation of four management units within the Missouri and Mississippi river basins: Great Plains Management Unit (GPMU), Central Lowlands Management Unit (CLMU), Interior Highlands Management Unit (IHMU) and Coastal Plains Management Unit (CPMU) (Jordan et al. 2016; USFWS 2014). The U.S. Fish and Wildlife Service (USFWS) implemented conservation augmentation programs that include the release of hatchery‐reared Pallid Sturgeon to bolster declining populations (Steffensen et al. 2012; USFWS 2014). Given the species' slow growth, delayed maturity and intermittent spawning, artificial propagation and population augmentation through stocking represent the primary short‐term strategy for sustaining Pallid Sturgeon populations in the lower Missouri River, where natural recruitment is largely absent (Steffensen et al. 2012). Potential broodstock are screened with microsatellite markers to ensure that they are pure Pallid Sturgeon and that they are not unmarked hatchery‐origin fish from previous stockings. These efforts are complemented by habitat restoration plans—such as reconnection of floodplain systems, improvement of water quality and modification of river channel structures—to reestablish natural spawning conditions and enhance recruitment success (USFWS 2014). Earlier studies emphasise that adaptive management, integrating genetic monitoring and continuous evaluation of habitat restoration outcomes is crucial to mitigate the impacts of hybridisation with Shovelnose Sturgeon and habitat fragmentation (Schrey et al. 2011; USFWS 2014). This multifaceted approach aims to preserve the genetic integrity and ecological function of Pallid Sturgeon populations, ensuring their long‐term viability in increasingly altered river systems (USFWS 2014).

Introgressive hybridisation, the incorporation of genetic material from one species into the genome of another through repeated backcrossing (Allendorf et al. 2001; Rhymer and Simberloff 1996), can profoundly erode the genetic integrity of populations, particularly among endangered species. A recent finding that all wild fish exhibiting Pallid Sturgeon morphologies from the CPMU are hybrids/backcrosses underscores the potential threat to the conservation of native gene pools (Jordan et al. 2019; Schrey et al. 2011; USFWS 2014). Hybridisation can occur as a natural process or can be enhanced by anthropogenic activities. The inadvertent inclusion of hybrid individuals in augmentation programs may dilute the unique genetic characteristics of native species, thereby compromising their adaptive potential and long‐term viability (Allendorf et al. 2001; Wolfe 2001). These challenges highlight the need for identifying genetically pure broodstock, ensuring the preservation of genetic diversity and the evolutionary resilience of endangered populations.

Pallid Sturgeon was initially recognised in 1905 as a distinct species from Shovelnose Sturgeon based on morphology (Forbes and Richardson 1905), a result that was amplified by subsequent studies (Bailey and Cross 1954; Kuhajda et al. 2007; Wills et al. 2002). The species also differ in diet (Carlson et al. 1985; Holley et al. 2022), age and growth (Carlson et al. 1985) and pigmentation (Kynard et al. 2002). The two species exhibit significant allele frequency differences where they occur in sympatry (Campton et al. 2000; Eichelberger et al. 2014; Flamio Jr. et al. 2025; Phelps and Allendorf 1983; Schrey et al. 2007; Tranah et al. 2004). While Pallid and Shovelnose sturgeons are clearly distinct species, they are genetically more similar to each other than many intraspecific animal populations (Allendorf et al. 2001; Campton et al. 2000). Historically, research has predominantly relied on allozymes, mitochondrial DNA sequences, microsatellites and a limited number of single nucleotide polymorphisms (SNPs) (Campton et al. 2000; Eichelberger et al. 2014; Phelps and Allendorf 1983; Schrey and Heist 2007). To date, no fixed genetic differences have been detected between the species, and identification of species and hybrids relies on statistical analyses based on multilocus genotypes. The recent RAD‐seq SNP study proved that the similar amount of genetic divergence between Pallid and Shovelnose sturgeon is far greater than differences between management units in either species (Flamio Jr. et al. 2025).

Currently, USFWS relies on a panel of 19 microsatellite markers (McQuown et al. 2000) for species identification, but these are insufficient for identifying pure species where hybrids and backcrosses occur (Jordan et al. 2019). Although recent advances, such as the application of double digest restriction site‐associated DNA sequencing (ddRAD‐seq), have expanded the number of loci that can be scored in non‐model species (Peterson et al. 2012), the resulting large panels are often cost‐prohibitive and unwieldy for routine conservation monitoring. Consequently, there is a need for a streamlined, fine‐scale marker panel that is more powerful than the current suite of microsatellite markers and balances high resolution with cost‐effectiveness in conservation genetics (Allendorf et al. 2010; Campbell et al. 2015; Shafer et al. 2015). Emerging methods, such as Genotyping‐in‐Thousands by Sequencing (GT‐seq), offer a promising solution by enabling targeted, high‐throughput SNP genotyping, which is particularly well‐suited for the management and recovery of endangered species like the Pallid Sturgeon (Campbell et al. 2015).

GT‐seq is an innovative, PCR amplicon‐based method that has recently gained prominence in conservation genetics and population genomics for its efficiency and cost‐effectiveness (Burgess et al. 2022; Campbell et al. 2015; Chang et al. 2021; Euclide et al. 2022; Harris et al. 2024; Schmidt et al. 2020). By enabling the simultaneous genotyping of hundreds of SNPs markers across hundreds or thousands of samples, GT‐seq leverages multiplex PCR in conjunction with high‐throughput sequencing platforms (Campbell et al. 2015). The high resolution afforded by GT‐seq enhances the accuracy of genotype calls and is especially valuable for detecting and monitoring introgressive hybridisation events. With a sufficiently large panel of markers, GT‐seq improves the capacity to accurately identify hybrids using model‐based statistical tools such as NewHybrids (Anderson and Thompson 2002). This increased resolution is critical for understanding the ecological and evolutionary consequences of hybridisation, as well as for informing management strategies aimed at preserving the genetic integrity of endangered species.

The current study extends the genetic marker repertoire available for population genetic analyses of *Scaphirhynchus*. *Scaphirhynchus* has undergone whole genome duplication and subsequent rediploidisation, resulting in approximately 120 chromosomes and a paleotetraploid but functionally diploid genome (Redmond et al. 2023; Vasil'ev 2009). This genomic complexity complicates the development of conventional diploid markers unless haploid individuals are sequenced (Flamio Jr. et al. 2025) or long‐read sequencing is applied. We leveraged a previously published ddRAD‐seq study utilising gynogenetic haploid Shovelnose Sturgeon, as well as ddRAD‐seq population data of adult Shovelnose and Pallid sturgeon, to mine informative SNP markers for the development of a novel GT‐seq panel (Flamio Jr. et al. 2025). Our newly developed GT‐seq marker panel outperforms conventional marker sets in species identification and parentage assignment and provides a robust, high‐throughput tool for monitoring genetic integrity and hybridisation dynamics in these imperilled species. Moreover, the enhanced resolution afforded by the GT‐seq approach is expected to facilitate more effective conservation management by enabling fine‐scale assessments of genetic diversity, structure and gene flow within and among *Scaphirhynchus* populations. This methodological advancement contributes to the broader field of conservation genomics by offering a scalable, cost‐effective solution for addressing the challenges posed by introgressive hybridisation and population decline in an imperilled taxa with closely related, and usually more abundant congener.

## 2 Methods

### 2.1 Sample Collection

For the pre‐design phase of GT‐seq marker panel, fin clip samples and sequence variants previously used to score *Scaphirhynchus* disomic markers (Flamio Jr. et al. 2025) were employed in the development and validation of a GT‐seq marker panel. That study comprised 120 individuals, including Pallid and Shovelnose sturgeon from the GPMU wild‐origin population and wild‐caught individuals from the CLMU, with initial species identification determined by microsatellite genotyping (Flamio Jr. 2022; Jordan et al. 2019; Steffensen et al. 2019). Six of the wild‐caught CLMU Pallid Sturgeon identified as F_1_ hatchery‐origin fish with GPMU parentage (Flamio Jr. et al. 2025) were reclassified as part of the GPMU sample; the remainder of the CLMU Pallid Sturgeon, and all the Shovelnose Sturgeon, were of wild origin. Detailed sample information is provided in Table S1 and Flamio Jr. (2022). For the post‐design phase—baseline construction, we incorporated 428 samples collected between 1992 and 2024 from the CLMU and GPMU. Samples were collected by multiple state and federal agencies working under permits provided by the U.S. Fish and Wildlife Service as part of the Pallid Sturgeon recovery program. Samples were collected as fin clips preserved in 95% ethanol stored at 4°C at Southern Illinois University Carbondale. Genomic DNA was isolated from fin clips using the QIAGEN DNeasy Blood & Tissue Kit (Qiagen, Hilden, Germany) and DNA solutions were stored at −20°C. Because Pallid and Shovelnose Sturgeon hybridise introgressively, it is impossible to know a priori which of the samples are pure Pallid or Shovelnose sturgeon. However, each of the 428 samples had been previously genotyped using microsatellites and exhibited a high posterior probability (> 95%) of being classified as either pure Pallid or Shovelnose Sturgeon (Table S2) and were checked against known hatchery crosses to rule out hatchery origin. These samples serve as valuable controls for validating the efficacy of the newly developed markers.

### 2.2 GT‐Seq SNP Panel Design

We used ddRAD‐seq data from 67 Pallid Sturgeon and 53 Shovelnose Sturgeon (Table S1; Flamio Jr. et al. 2025) for GT‐seq SNP marker development. The data processing of SNP calling and filtering is followed in Flamio Jr. et al. (2025) which was derived from an established protocol (Flamio Jr. 2022; Flamio Jr. et al. 2025; O'Leary et al. 2018). It mainly categorised into three steps: (1) demultiplex NGS data by Stacks v2.0 (Rochette et al. 2019); (2) Trim and map reads and call SNPs using dDocent (Puritz et al. 2014); (3) SNP filtering (details of criteria quality scores can be found in Supporting Information). Samples were assigned to the GPMU and CLMU populations based on collection location, and parentage analysis was conducted to identify hatchery‐origin offspring among wild‐caught CLMU individuals (Flamio Jr. et al. 2025; Kalinowski et al. 2007; Marshall et al. 1998). Such individuals identified as offspring from known GPMU stockfish lineage were either removed or reclassified as part of the GPMU population. Each population was then tested for Hardy–Weinberg equilibrium using a Perl script nested in dDocent (https://github.com/jpuritz/dDocent/blob/master/scripts/filter_hwe_by_pop.pl) (Puritz et al. 2014). The final SNP set is 29,593 variants.

To minimise linkage bias, only one SNP per contig was retained. Selected loci were anchored on a partial linkage map (Flamio Jr. 2022), and loci pairs situated within 50 centimorgans on the same linkage group were excluded. For development of a specialised marker panel for species identification and parentage analysis, filtered SNPs from the Pallid Sturgeon dataset were further analysed to estimate genetic divergence (F_ST_) (Weir and Cockerham 1984) and nucleotide diversity (Nei and Li 1979)—same as expected heterozygosity in SNP, using VCFtools v0.1.14 (options “—weir‐fst‐pop” and “—site‐pi”) (Danecek et al. 2011).

Two sets of SNP panels were created based on ranked value: one based on F_ST_ between the species to identify loci with strong discriminatory power for species identification (S‐loci) and the other based on nucleotide diversity within Pallid Sturgeon to select loci with high polymorphism for population analysis (P‐loci). In total, 291 P‐loci and 303 S‐loci, each flanked by at least 15 bp on both sides of the target SNP, were selected for GT‐seq primer design. Candidate loci were subsequently submitted to GTseek (https://gtseek.com/) for GT‐seq marker development, leveraging their expertise in designing primers for multiplex PCR reactions.

### 2.3 GT‐Seq Primer Design

Primers were designed using a proprietary pipeline optimised for the development of multiplex PCR‐compatible primer pools. Briefly, primers were selected to have melting temperatures between 58°C and 64°C, excluding the R1 and R2 tag sequences. Each locus‐specific primer pair was designed to generate amplicons between 80 and 150 base pairs in length, with the target SNP site located within the first 75 bases of the amplicon.

For each SNP locus, the pipeline generated up to 40 candidate primers for both the left and right flanks. These candidates were then screened against previously accepted primers to identify potential cross‐binding sites that could lead to primer‐dimer artefacts. Specifically, any candidate primer for which the last 10 bases exhibited an annealing temperature above 15°C to a previously accepted primer was excluded. Primers were also rejected if they were predicted to form stable hairpin structures with melting temperatures above 50°C.

All primers were synthesised by Integrated DNA Technologies (IDT; https://www.idtdna.com/) at 200 μM in TE buffer (pH 8.0). The initial primer pool for testing was made by preparing a primer mix using 2 μL of each primer.

### 2.4 GT‐Seq Library Preparation

DNA was extracted from fish fin clips using the QIAGEN DNeasy Blood & Tissue Kit (Qiagen, Hilden, Germany). The extracted DNA was quantified using a Qubit 4 Fluorometer (Thermo Fisher Scientific, Waltham, Massachusetts, USA) and the 1× dsDNA HS Assay Kit (Thermo Fisher Scientific, Eugene, Oregon, USA). Only samples with DNA concentrations exceeding 10 ng/μL were used for downstream processing; samples failing this threshold were re‐extracted when additional tissue was available.

Library preparation followed the protocol of Campbell et al. (2015), with a modification to dilute the PCR1 product to a 1:10 ratio, as described by Chang et al. (2022). To accommodate primers with melting temperatures above 60°C, the annealing temperature was increased to 60°C and the annealing step was extended to 2 min. Given the short target amplicon length of approximately 100 bases, the extension time was reduced to 30 s. The PCR2 products were then pooled and normalised using Nate's Plates 96‐well Plate Kit (GTseek LLC, Twin Falls, Idaho, USA), followed by additional PCR to amplify the normalised amplicons. The final products underwent size selection and purification using SPRIselect beads (Beckman Coulter, Brea, California, USA) and were eluted in a final volume of 15 μL.

The library was quantified using the NEBNext Library Quant Kit (New England Biolabs, Ipswich, Massachusetts, USA) for Illumina. A 20 pM library was prepared and loaded into the MiSeq Reagent Kit v3 (150 cycles) (Illumina, San Diego, California, USA) for 75 bp paired‐end sequencing on an Illumina MiSeq platform at the Conservation Genomics Laboratory of Southern Illinois University Carbondale.

### 2.5 GT‐Seq Genotyping and Primer Optimisation

Of the 120 samples, 85 samples (38 pallid sturgeons and 47 shovelnose sturgeon) (Table S3) with > 80% genotypes from both ddRAD‐seq and GT‐seq data were used to examine the concordant pattern of genotype. The ddRAD‐seq genotypes are available in Flamio Jr. (2022). The GT‐seq pipeline (https://github.com/GTseq/GTseek_utils) was used to process raw sequencing data and call genotypes. This pipeline included demultiplexing, counting primer and allele occurrences and genotype calling based on the default ratio determination with a minimum sequencing depth threshold of 10× described by Campbell et al. (2015). The occurrence of on‐target forward and reverse reads, matched probe sequence reads and matched pair reads were counted using the script “GTseq_PrimerTest.pl” from the GTseek_utils GitHub repository (https://github.com/GTseq/GTseek_utils/blob/Main/GTseq_PrimerTest.pl). This allowed screening of low efficiency primers to be removed in multiplex PCR.

Three rounds of primer optimisation were performed: (1) removal of primers associated with overamplification, primer artefacts and off‐target sequences and elimination of potential tetrasomic loci and repetitive loci, which exhibited complex allelic ratio patterns inconsistent with disomic expectations (i.e., three expected ratio patterns, where ~1 indicates heterozygosity and values near 0 or high extremes indicate homozygosity); (2) exclusion of loci with > 5% discordant genotype between ddRAD‐seq and GT‐seq; and (3) improvement of amplification efficiency by removing primers with low on‐target rates and poor agreement between forward and reverse primers.

Our newly developed GT‐seq panel includes two sub‐panels: S‐loci for species identification and P‐loci for parentage analysis. For this study, we used Cervus v3.7 (Kalinowski et al. 2007) to perform parentage assignments, which are based on observed allele and genotype frequencies to estimate the likelihood of parent‐offspring matches. To reduce bias in assignment results, we removed P‐loci with high missing data or significant deviations from Hardy–Weinberg equilibrium, as these can artificially inflate heterozygosity estimates. The remaining loci were used for downstream simulations.

### 2.6 Power Analysis

#### 2.6.1 Baseline Compilation

All 428 Pallid and Shovelnose Sturgeon baseline samples previously curated using 19 microsatellites and validated through STRUCTURE (Pritchard et al. 2000) and NewHybrids (Anderson and Thompson 2002) were collected for constructing the GT‐seq baseline dataset (Jordan et al. 2019). These samples were further revalidated in this study using NewHybrids. Option “z” was used to designate known groups based on previous microsatellite assignment and agreement with morphological identification. Individuals with species assignment probabilities below 95% based on microsatellite data or below 99% based on GT‐seq data were excluded from the final baseline database. This curated dataset was then used for genotype simulation and power analysis.

Each sample was assessed for species assignment accuracy, ensuring robust identification of Pallid and Shovelnose sturgeon individuals. Genotype data were first converted to Genepop format (Rousset 2008) and subsequently reformatted for NewHybrids (Anderson and Thompson 2002) using the genepopedit R package (Stanley et al. 2017). NewHybrids v2.0 (Linux command‐line version) was used for species assignment under the Jeffreys prior for both theta and pi parameters, with a burn‐in of 10,000 iterations followed by 100,000 sweeps (Anderson and Thompson 2002). Analyses were conducted using fixed random seed values (100 and 200) and the default two‐generation hybrid model.

To streamline computation for large datasets, we employed the parallelnewhybrid R package (Wringe et al. 2017b), specifically the parallelnh_LINUX function, allowing for parallel execution of NewHybrids analyses across multiple processors using the default settings (burn‐in 2000 and sweeps 10,000).

#### 2.6.2 Baseline Database Imputation for Power Test

Uneven distributions of missing data across loci or individuals can distort simulated genotype outcomes and downstream analyses. Therefore, imputation was performed to standardise data completeness. The original baseline database has incomplete genotypes across samples, in both the 19‐locus microsatellite dataset (McQuown et al. 2000) and GT‐seq SNP dataset. Allele and genotype frequencies were calculated using a custom R script (imputation.R, https://github.com/JunmanHuang/rscript‐gtseq‐scaphirhynchus) (R Core Team 2024). Missing genotypes were imputed using the most frequently observed genotype at each locus within the reference population. The imputed dataset was used exclusively for simulation‐based power analyses and not for empirical applications.

#### 2.6.3 Species and Hybrid Classification

We evaluated the accuracy, efficiency and power of hybrid classification using NewHybrids (Anderson and Thompson 2002) through simulation‐based analyses. For each of six hybrid categories within two generations—pure Pallid Sturgeon (Pal), pure Shovelnose Sturgeon (Sho), F1, F2 and F1 backcrosses to Pallid Sturgeon (B × P) and F1 backcrosses to Shovelnose Sturgeon (B × S), we simulated 30 individuals per class using the freqbasedsim_GTFreq function (Wringe et al. 2017a), applied to four different marker panels: P‐loci, S‐loci, the full GT‐seq SNP dataset and the 19‐locus microsatellite panel. Simulations and hybrid classification assessments were conducted using the hybridpowercomp function implemented in the R package hybriddetective (Wringe et al. 2017b). Each simulation scenario was replicated three times.

To investigate the effect of missing data on assignment accuracy, one replicate simulation from each marker panel was selected. We developed a custom R script (simNA_plot.R, https://github.com/JunmanHuang/rscript‐gtseq‐scaphirhynchus) to introduce controlled levels of missing data, ranging from 0% to 90% into the existing genotype matrix. These modified datasets were then used as input for NewHybrids, employing the same model settings, to evaluate changes in posterior probability assignment under varying levels of data completeness.

#### 2.6.4 Parentage Assignment

To evaluate the power of the marker panel for distinguishing hatchery‐ and wild‐origin offspring through parentage analysis, we simulated offspring genotypes using known broodstock genotypes. Because not all baseline individuals had known sex or broodstock status, we retained only those present in both the baseline and broodstock datasets (Table S4).

Simulations were performed in Cervus v3.7 (Kalinowski et al. 2007), generating 10,000 simulated offspring from 100 candidate males and 100 candidate females, with a 100% parental sampling rate (see Table S5 for detailed simulation parameters). Parentage assignment accuracy was assessed by calculating the proportion of correctly assigned parents. We also computed the Delta score (the difference between the likelihood ratio scores (LOD) of the most and second most likely parents), which reflects assignment confidence—higher values indicate greater confidence. Simulations were conducted under both 80% (relaxed) and 95% (strict) confidence thresholds to illustrate the trade‐off between accuracy and certainty.

To assess the discriminatory power of different marker panels, we conducted a sensitivity analysis using consistent locus parameters: 90% of loci typed, 2% genotyping error (mistyped loci), and minimum typed loci thresholds set at 50%, 60%, 70% and 80% (Table S5). This analysis compared two marker panels (19 microsatellites and P‐loci) for rapid examination. To evaluate the effect of missing genotype data, simulations were repeated for four marker panels (microsatellites, P‐loci, S‐loci and the full GT‐seq SNP panel) with five levels of missing data (10%–50%), using a fixed threshold of 50% minimum typed loci. To examine the influence of parental sampling proportion on assignment accuracy in the P‐loci panel, we tested the proportion of sampled parents from 40% to 100%. Assignment rates and critical Delta values were visualised as line plots using the ggplot2 package in R (Wickham 2016).

## 3 Results

### 3.1 GT‐Seq SNP Panel Design

**FIGURE 1:**
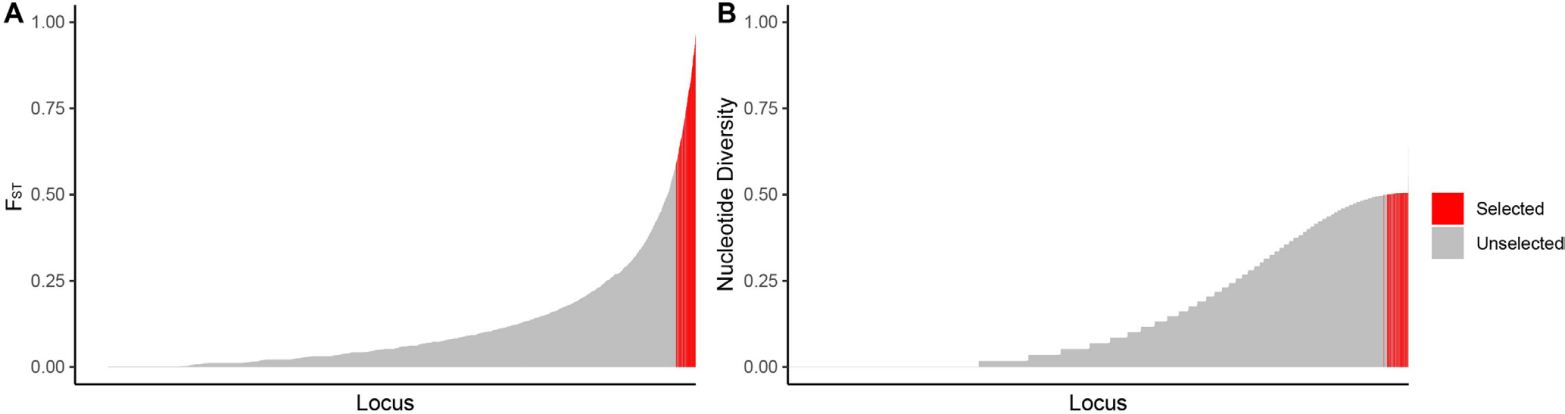
Distribution of genetic variation across loci in the initial SNP dataset (~29,000 loci), ordered by increasing values. Red bars indicate loci selected for GT‐seq marker development. (A) A total of 303 loci were selected based on high genetic divergence (FST) between Pallid Sturgeon ( *S. albus* ) and Shovelnose Sturgeon ( *S. platorynchus* ). (B) The top 291 loci with high nucleotide diversity within Pallid Sturgeon were selected.

Sequencing data generated by Flamio Jr. (2022) for 120 *Scaphirhynchus* individuals were used to extract SNP genotypes. Samples from different management units were treated as separate populations for equilibrium filtration. The dataset consisted of 44 GPMU Pallid Sturgeon, 23 CLMU Pallid Sturgeon, 29 GPMU Shovelnose Sturgeon and 24 CLMU Shovelnose Sturgeon (Table S1). Following preliminary SNP filtration, including filtration via Hardy–Weinberg equilibrium and linkage equilibrium testing within species, the initial SNP panel contained 29,593 SNPs. 594 loci, including the top 303 loci with high F_ST_ values and 291 loci with high nucleotide diversity, were selected for GT‐seq primer design through GTseek Inc. Their distribution of the selected loci among other candidate loci is depicted in Figure 1.

### 3.2 GT‐Seq Genotyping and Primer Optimisation

An initial test of primer efficiency was conducted on 96 samples, 85 of which overlapped with those previously used in ddRAD‐seq and 11 newly tested samples for baseline construction. Loci were excluded if they exhibited low on‐target read percentages, signs of tetraploidy, abnormal allele ratios, or lacked corresponding ddRAD‐seq data. After these filters, 356 loci were retained (Figure S1).

**FIGURE 2:**
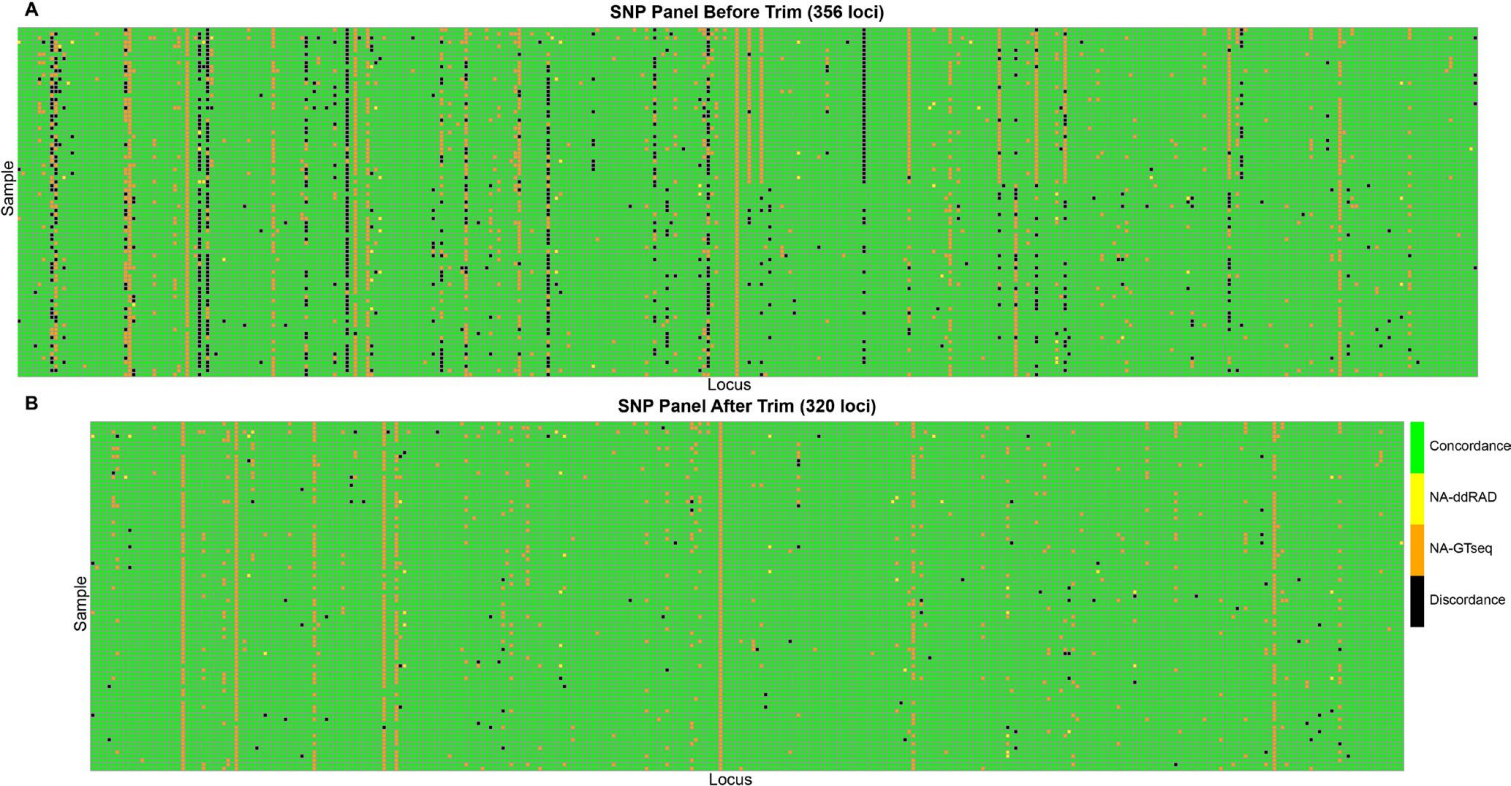
Heatmap illustrates genotype concordance between ddRAD‐seq and GT‐seq data for both unfiltered and filtered SNP datasets. Each row represents an individual sample (*n* = 85), and each column corresponds to a GT‐seq SNP locus. Colour coding: Green = concordant genotypes; yellow = missing genotype in ddRAD‐seq, orange = missing genotype in GT‐seq, black = discordant genotypes between the two methods.

For concordance evaluation, genotypes from the 85 samples genotyped using both ddRAD‐seq and GT‐seq were compared across the 356 loci. The ddRAD‐seq dataset had at least 98% genotypes while GT‐seq had at least 88% genotypes. Concordance scoring assignments were defined as follows: (1) 2 when both methods produced identical genotypes; (2) 1 when only the ddRAD‐seq genotype was missing; (3) 0 when only the GT‐seq genotype was missing; (4) −1 when both genotypes were missing; and (5) −2 for discordant genotypes. To ensure high concordance, loci were retained only if the number of discordant genotypes (score of −2) was fewer than five (i.e., less than 5% of 85 samples). Based on this threshold, 320 loci passed the concordance filter (Figure 2). Three loci deemed non‐informative—such as those with no observed genotype variation across all samples or consistent missing data—were also excluded. Subsequently, an additional 18 loci were removed based on primer agreement rate (probe sequence from forward read match to reverse read) < 50% among four datasets (Table S3) to further optimise multiplex PCR efficiency.

Because parentage analysis requires Mendelian markers and is very sensitive to genotyping errors, we removed 32 loci that either exhibited significant excess heterozygosity or had high levels of missing data (Tables S18–S21). We retained 112 P‐loci for parentage analysis and simulations for the P‐loci. The S‐loci panel remained 155 loci. Altogether, the final GT‐seq panel set included 267 loci (probe sequence see Table S6 and primer sequence see Table S7).

### 3.3 Baseline Compilation

#### 3.3.1 Baseline Imputation for Simulation

**FIGURE 3:**
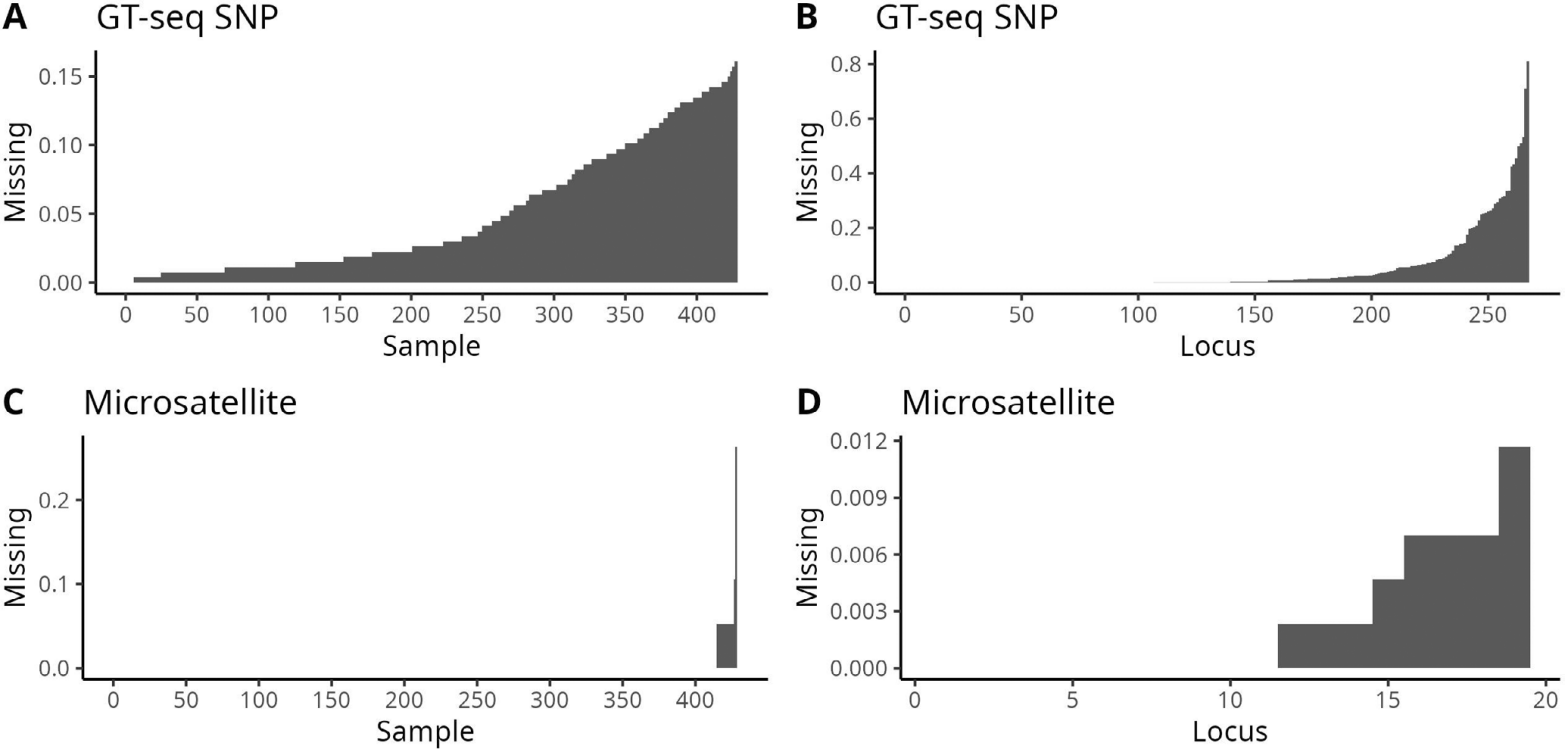
Missing data proportions at the sample and locus levels across all baseline samples (*n* = 424) and loci. Panels A and B present the characteristics of the GT‐seq SNP panel, while panels C and D show the corresponding patterns for the 19 microsatellites panel. The *x*‐axis represents the index number of either the samples or the locus, and the *y*‐axis indicates the proportion of missing data.

In total, 428 Pallid and Shovelnose Sturgeon baseline samples, previously curated in microsatellite data through STRUCTURE and NewHybrids validation (Jordan et al. 2019), were reanalysed to assess species assignment resolution between microsatellite and GT‐seq panels. NewHybrids was rerun separately for each marker panel, and individuals with posterior probabilities < 95% in microsatellite panel or < 99% GT‐seq panel were excluded from downstream simulations. Four samples were removed: two from the microsatellite panel (354‐012 and 354‐013) and two from the GT‐seq panel (MOS‐629 and MOS‐788), resulting in 424 high‐confidence baseline samples (Tables S8 and S9). The missing percentage of each locus is shown in Figure 3 (plotted from script ‘meta_baseline.R’, https://github.com/JunmanHuang/rscript‐gtseq‐scaphirhynchus). Missing data patterns are summarised in Figure 3 (generated with ‘meta_baseline.R’). While locus‐level missingness was comparable across panels, sample‐level missingness was higher in the GT‐seq SNP panel: over half of the samples had > 5% missing genotypes compared to microsatellites (Figure 3A,C). At the locus level, both panels had over half of the loci with minimal missing data (Figure 3B,D). The allele and genotype frequencies for each locus on various marker panels and species groups can be viewed in Tables S10–S17. The most frequent genotype for each locus was used to replace the missing data for simulation tests in hybrid detection and parentage analysis.

### 3.4 Species Identification

**FIGURE 4:**
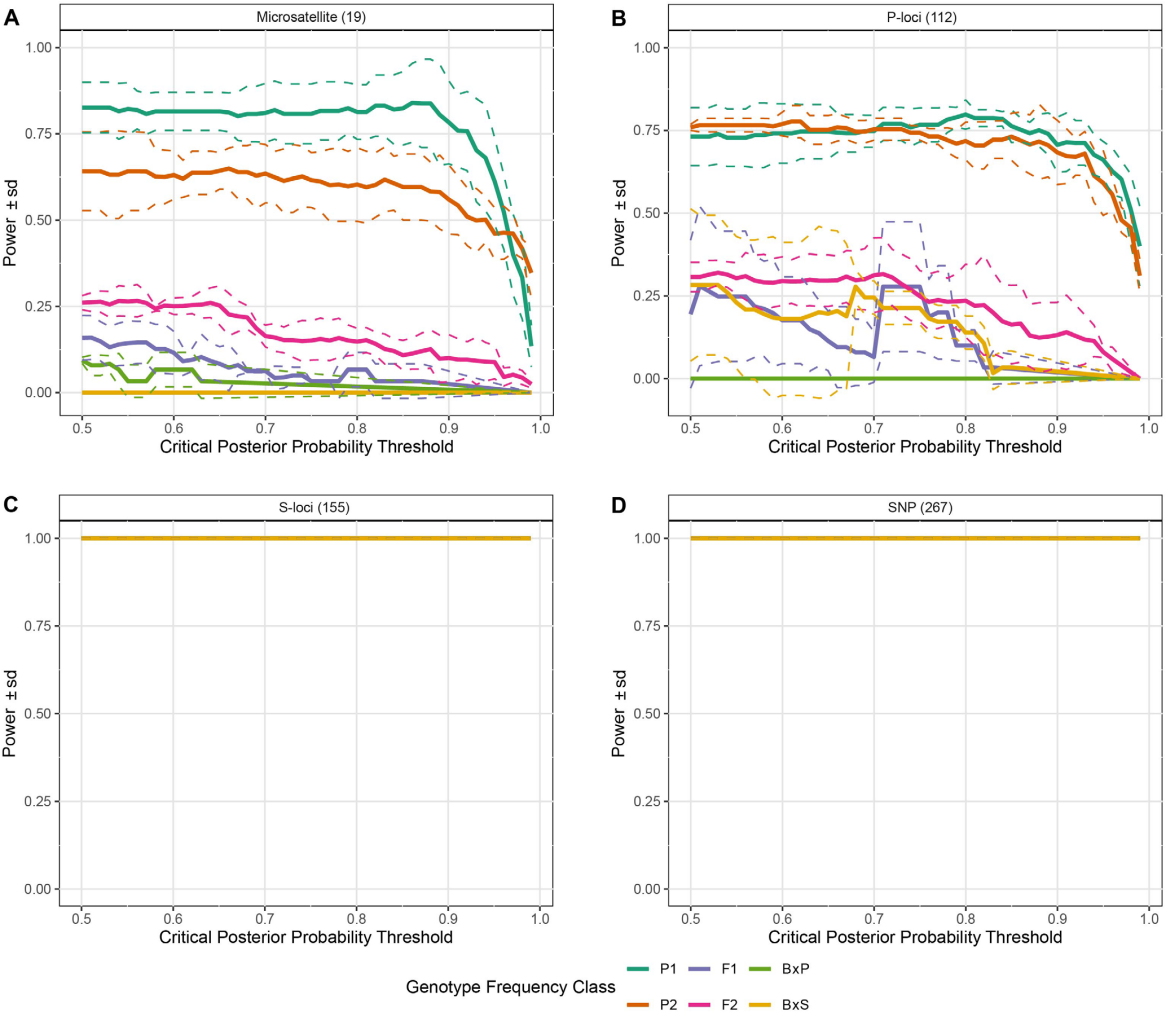
Power analysis results from the *Hybriddetective* R package, which was used to assess classification accuracy across posterior probability thresholds (0.5–1.0). Species and hybrids categories are indicated by different line colours; later categories are overlaid on earlier ones. Solid lines represent mean power, and dotted lines indicate the standard deviation across three independently simulated datasets, each analysed in triplicate. Panels A and B show that 19‐microsatellite and 112‐SNP (P‐loci) panels exhibit moderate power in distinguishing pure species and hybrids categories, but perform poorly in detecting backcrosses. Panels C and D demonstrate that the 155‐SNP (S‐loci) and 267‐locus GT‐seq SNP panels provide higher resolution and more reliable classification of all hybrid categories. Categories: Pal = Pure Pallid Sturgeon ( *S. albus* ); Sho = Pure Shovelnose Sturgeon ( *S. platorynchus* ); B × P = Backcross to Pallid Sturgeon; B × S = Backcross to Shovelnose Sturgeon.

Allele frequencies from 424 curated baseline samples (249 Pallid and 175 Shovelnose Sturgeons) served as references to simulate 30 individuals for each hybrid category (Pal, Sho, F_1_, F_2_, B × P, B × S). The newly developed GT‐seq SNP markers demonstrated strong discriminatory power in differentiating hybrid categories and achieved 100% accuracy across all posterior probability thresholds (Figure 4). The conventional 19 microsatellite markers showed moderate accuracy (60%–80%) in identifying simulated pure species but performed poorly in identifying simulated backcross hybrids (Figure 4A). To assess the individual power of the P‐loci and S‐loci sub‐panels for species assignment, we performed separate analyses using NewHybrids. The P‐loci panel showed similar performance to the microsatellite panel, moderately assigning simulated individuals to pure species categories, but weakly to hybrids categories (Figure 4B). However, the S‐loci panel alone maintained the strong performance level observed with the full 267‐SNP panel (Figure 4C,D).

**FIGURE 5:**
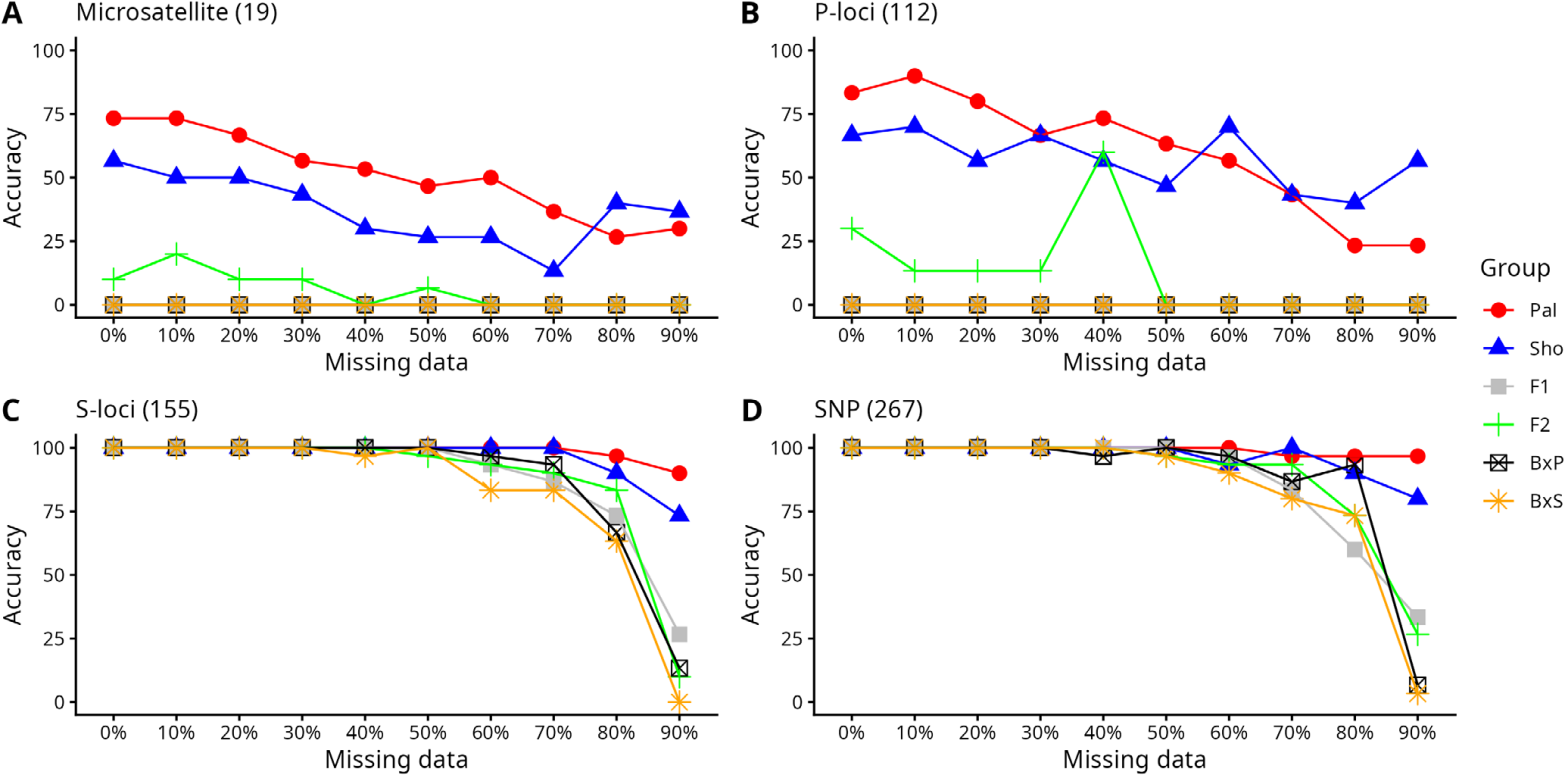
Assignment accuracy of simulated individuals to the correct hybrid categories at varying levels of missing data (0%–90%), using a > 90% posterior probability threshold. Each hybrid category group consisted of 30 simulated individuals; only pure parentals were designated with z‐score for highly accurate classification in NewHybrids. Panel A shows results for the 19‐locus microsatellite panel, panel B for the P‐loci panel (112 SNPs), panel C for the S‐loci panel (155 SNPs) and panel D for the complete SNP dataset (267 SNPs). Categories: Pal = Pure Pallid Sturgeon ( *S. albus* ); Sho = Pure Shovelnose Sturgeon ( *S. platorynchus* ); B × P = Backcross to Pallid Sturgeon; B × S = Backcross to Shovelnose Sturgeon.

To determine how missing data affects the accuracy of species identification, we examined the performance of different marker panels at varying levels of missing data (Figure 5). The S‐loci panel and the full SNP panel (all loci) maintained high accuracy (90% resolution) for species identification until 60% of the data was missing (Figure 5C,D). In contrast, the P‐loci and microsatellite panels showed only moderate resolution (80%) in identifying pure species even with complete genotype data (Figure 5A,B). The P‐loci consistently maintained moderate resolution (50%–80% accuracy) for pure species identification up to 60% missing data, whereas the microsatellite panel began to lose moderate resolution, maintaining around 30%–75% accuracy. Notably, both the P‐loci and microsatellite panels had limited ability to differentiate hybrids across all levels of missing data.

### 3.5 Parentage Assignment

**FIGURE 6:**
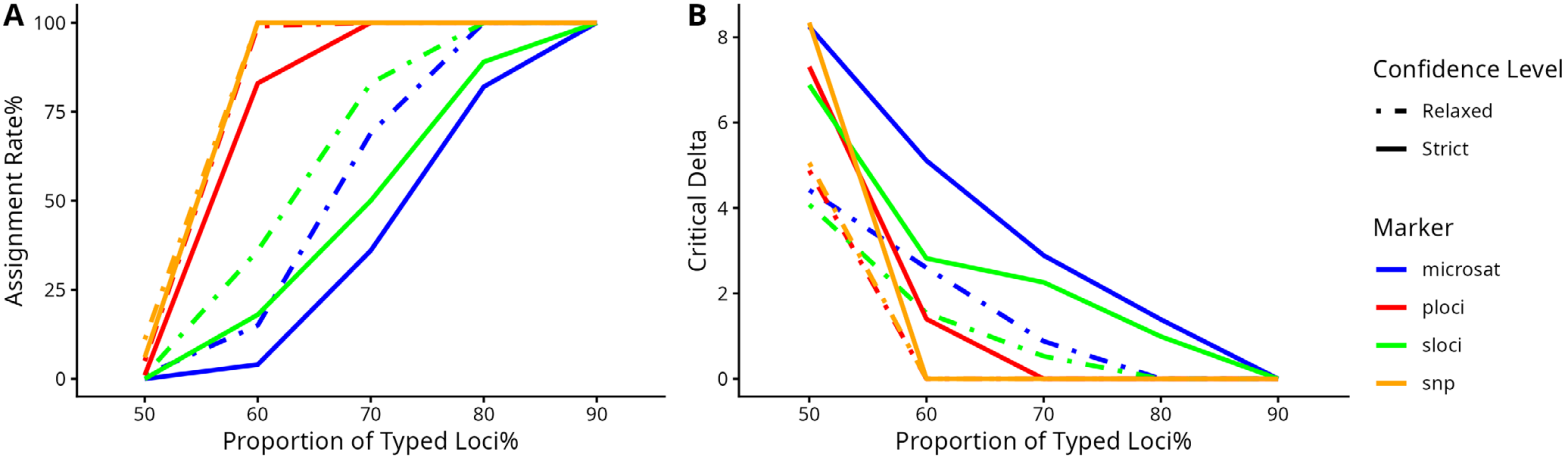
Sensitivity analysis of simulated parentage assignment in Cervus (Kalinowski et al. 2007) under varying proportions of typed loci across multiple marker panels (indicated by line colour). Analyses were conducted at two confidence levels: Relaxed (80%, dot‐dashed lines) and strict (95%, solid lines). Panel A presents assignment rates, representing the proportion of offspring correctly assigned to their true parents. Panel B shows critical Delta values, indicating the confidence of parentage assignment. Marker panels: Microsat = microsatellite, p‐loci = parentage assignment panel, s‐loci = species identification panel, snp = GT‐seq SNP panel including p‐loci and s‐loci.

We used 126 baseline individuals (83 males and 43 females) (Table S4), which were used as broodstock, to simulate parentage assignment with known parental sex. To assess the impact of missing data on parentage analysis, we fixed the minimum typed loci threshold at 50% and varied the proportion of typed loci from 50% to 90% across marker panels. All panels showed increased assignment rates with higher proportions of typed loci, but the patterns differed among marker types. For P‐loci and the full SNP panel, assignment rates (around 60%–70% proportion of typed loci) approached an asymptote earlier compared to the rest of panels. In contrast, the microsatellite and S‐loci panels showed an S‐shaped curve and reached 100% assignment rates at 90% proportion of typed loci. Overall, P‐loci and the full SNP panel consistently outperformed the microsatellite and S‐loci panels in assignment accuracy (Figure 6A). Delta scores for P‐loci and SNP panel declined as typed loci increased and rapidly dropped to zero at 60%–70% typed loci, indicating reduced assignment resolution at higher data completeness. While Delta values for microsatellites and S‐loci declined more gradually (Figure 6B). These trends were consistent under both relaxed (80%) and strict (95%) confidence thresholds.

To evaluate the effect of minimum typed loci, we fixed the proportion of typed loci at 90% and varied the minimum typed loci from 50% to 80%. Assignment rates for P‐loci remained consistently high across all thresholds, while those for microsatellites began to decline at 70% minimum type of loci. Conversely, critical Delta values for P‐loci remained stable at zero, whereas values for microsatellites increased beyond the 70% threshold (Figure S2A,C).

To isolate the effect of parental sampling proportion, we fixed the proportion of typed loci at 90% and the minimum typed loci at 50%. Varying the proportion of sampled parents from 40% to 100% revealed a linear increase in assignment rates (Figure S2B), while critical Delta values declined with higher sampling proportions (Figure S2D). These patterns were consistent across both confidence levels.

Among the 126 Pallid Sturgeon broodstock samples, the mean observed heterozygosity (H_o_) and expected heterozygosity (H_e_) values were comparable within each marker panel (Table 1). Microsatellites showed the highest mean observed heterozygosity (~0.64), followed by P‐loci (~0.50), while S‐loci and the full SNP panel were substantially lower (~0.11 and ~0.27, respectively)—representing six‐ and two‐fold reductions compared to microsatellites. Locus‐specific heterozygosity values were provided in Tables S18–S21. Overall, P‐loci and microsatellite panels exhibited higher heterozygosity, consistent with the design of P‐loci to maximise genetic variability for parentage analysis.

**TABLE 1:** Heterozygosity metrics of 126 Pallid Sturgeon ( *S. albus* ) broodstocks based on four marker panels.

Panel	H_o_ mean	H_e_ mean	Marker number
P‐loci	0.500	0.493	112
S‐loci	0.119	0.117	155
SNP	0.279	0.275	267
Microsatellite	0.642	0.646	19

## 4 Discussion

Accurate species identification in the presence of hybridisation, along with distinguishing hatchery‐origin from wild individuals, is crucial for the effective conservation of declining fish populations. In this study, we developed the first dual‐purpose GT‐seq panel for *Scaphirhynchus* sturgeons, capable of both high‐resolution species and hybrid classification—including backcrosses—and robust parentage assignment to differentiate hatchery from wild‐origin individuals. Simulation analyses demonstrated that this novel GT‐seq panel consistently outperformed the traditional microsatellite panel in both species identification and parentage assignment. This tool provides an efficient and scalable approach for monitoring the genetic outcomes of augmentation programs and supports informed management decisions for Pallid Sturgeon conservation.

### 4.1 GT‐Seq Panel Development and Evaluation in Species Identification

Developing GT‐seq markers for *Scaphirhynchus*, a genus with polyploid ancestry, is challenging because it went through ancient whole‐genome duplication followed by rediploidisation (Redmond et al. 2023; Vasil'ev 2009). As a result, duplicated (tetrasomic) and rediploidised (disomic) loci coexist, leading to genotypic ambiguity if these loci are not properly distinguished. Using standard short‐read sequencing approaches such as RAD‐seq or shotgun sequencing, it is difficult to reliably discriminate between tetrasomic and disomic loci. However, pre‐sequencing strategies that generate haploid gynogens—containing only a single parental genome—have proven effective for identifying and excluding tetrasomic loci, even when using short‐read data (Flamio Jr. et al. 2025). In addition, the increasing availability of long‐read sequencing technologies provides a complementary approach for resolving duplicated regions, thereby facilitating GT‐seq marker development in polyploid taxa.

Traditionally, GT‐seq has been applied primarily to disomic SNPs due to their straightforward genotyping and low sequencing cost. More recently, GT‐seq panels have expanded to include microhaplotypes, defined as sets of tightly linked SNPs inherited together, which offer increased resolution for population genetic and parentage analyses. Accordingly, existing GT‐seq SNP panels can be readily extended toward microhaplotype‐based designs (Anderson et al. 2025; Delomas et al. 2023; Thompson et al. 2025). In polyploid systems, however, genotype calling requires more stringent thresholds, and higher sequencing depth is often necessary because allele dosage differences among genotypes are more subtle at higher ploidy levels (Delomas et al. 2021). Despite these challenges, recent studies have successfully developed amplicon‐based sequencing panels for polyploid species and demonstrated their utility in conservation genetics (Delomas et al. 2021; Johnson et al. 2026).

The NewHybrids program operates under the assumption of unlinked markers for accurate species and hybrid identification. Including linked loci can artificially inflate posterior probabilities (Anderson et al. 2024). Our new GT‐seq panel includes 267 loci, and given that *Scaphirhynchus* has ~120 chromosomes (Fontana et al. 2001; Havelka et al. 2011), the number of markers exceeds chromosome number, potentially indicating the presence of physically linked loci located on the same chromosome arm. Currently, there is no complete genome assembly for *Scaphirhynchus* spp. to quantify the physical distance between loci on the same chromosome. Despite this potential for linkage, our simulations of missing data from both the full SNP panel and the S‐loci panel demonstrate that even with up to 70% missing data (leaving approximately 47 informative loci from the S‐loci panel), the power to distinguish pure Pallid Sturgeon and Shovelnose Sturgeon remains high, surpassing the performance of traditional microsatellite markers. This suggests that if future genome sequencing reveals close physical linkage between any two of our markers, we could remove one out of each pair of physically linked markers without significantly eroding the power of species identification. It implies that even with a future chromosome‐level genome assembly to rule out linked markers, a significant portion (around 30%) of our current GT‐seq panel will retain its effectiveness for distinguishing these two species.

### 4.2 Marker Evaluation in Parentage Assignment and Management Implications

Heterozygosity and marker number are critical factors determining the power of genetic markers for parental exclusion (Allendorf et al. 2022; Kathiravan et al. 2012; Labuschagne et al. 2015). Marker panels with lower heterozygosity (e.g., SNPs with H_e_ = 0.2) generally require three to five times more loci than high‐heterozygosity markers (e.g., microsatellites with H_e_ = 0.6) to achieve comparable power (Allendorf et al. 2022). By selecting SNPs with high H_e_, we increased the power of the P‐loci for parentage assignment above what would be expected for randomly selected SNPs. In terms of marker quantity, the novel GT‐seq panels included more loci than 19 microsatellites (McQuown et al. 2000): approximately 6–8× more for P‐loci and S‐loci, and 14× more for the full SNP panel. This increased density compensates for the lower allelic diversity typical of bi‐allelic SNPs. Our comparative evaluation of high‐polymorphism loci (P‐loci), high genetic divergence loci (S‐loci), the full SNP panel and traditional microsatellites revealed that both the GT‐seq panel and the P‐loci sub‐panel outperformed the S‐loci and microsatellites in parentage assignment accuracy (Figure 6A,B). These findings reinforce that marker informativeness—measured by both heterozygosity and number—is essential to optimise parentage assignment power in conservation genetics (Allendorf et al. 2022; Kathiravan et al. 2012; Labuschagne et al. 2015).

Assignment rate, defined as the proportion of offspring correctly assigned to their true parents, increased with the proportion of typed loci across all marker panels (Figure 6A). Notably, P‐loci and the full SNP panel rapidly reached an assignment rate plateau at 100% (Figure 6A). As Cervus cannot assign a true parent that is absent from the candidate pool, increased sampling directly improved assignment success (Figure S2B). Conversely, critical Delta values declined as more loci were typed, reflecting clearer signal and reduced evidence required to reach high‐confidence assignments (Figures 6B and S2D).

Marker panel size also influenced performance under stricter thresholds. In the microsatellite panel, assignment rates declined when the minimum typed loci threshold exceeded 70% (Figure S2A). It is because with only 19 markers available, increasing the threshold excluded more individuals from analysis, limiting assignment potential. This limitation was not observed in larger SNP‐based panels.

Overall, our results highlight the potential of SNP‐based panels for conservation management. For species and hybrid classification, the S‐loci provided high resolution not only for distinguishing parental species and early‐generation hybrids, but also for detecting introgressive backcrosses that retain a high proportion of parental genetic ancestry. Recovery of Pallid Sturgeon where natural recruitment is absent or insufficient, relies on a conservation stocking program that utilises wild‐caught broodstock spawned in hatcheries to produce offspring for stocking. Accurate identification of broodstock is critical, as misclassifying and stocking backcross Pallid Sturgeon could undermine long‐term conservation goals. Hatchery supplementation often produces far more surviving offspring per parent than natural reproduction, increasing the risk of genetic swamping and contamination of wild gene pools if hybrids are inadvertently propagated. Species identification is also important for ongoing surveys of wild‐spawned age‐0 sturgeon (Gosch et al. 2025). The ability to distinguish between Pallid, Shovelnose and hybrid (including backcrosses) sturgeon larvae and free embryos is critical for monitoring natural reproductive success in context with stocking to increase adult population sizes, habitat modifications designed to increase spawning habitat and age‐0 survival (DeLonay et al. 2009, 2016; Gosch et al. 2015), hybridisation and manipulations of seasonal releases of water from reservoirs to augment spawning cues (DeLonay et al. 2009). The P‐loci will also be useful for monitoring effective population size (N_e_) and effective numbers of breeders (N_b_) in a season via linkage‐based estimators (Do et al. 2014). Recovery criteria for Pallid Sturgeon under the U.S. Endangered Species Act are based in part on sufficient N_e_ in each management unit (USFWS 2014) and having a large number of markers increases precision of N_e_ and N_b_ estimates. Introgressive hybridisation is a threat to the persistence of Pallid Sturgeon (USFWS 2014) and the markers described in this study are the only tools currently available to reliably distinguish between pure and backcrossed Pallid Sturgeon for multiple conservation priorities including monitoring and propagation. For parentage analysis, the P‐loci panel substantially enhanced assignment power and, when combined with traditional microsatellites, can improve estimates of relatedness beyond simple parent–offspring relationships, including half‐sibling inference. Parentage analysis is used to distinguish between wild‐ and hatchery spawned Pallid Sturgeon adults with the former preferred for broodstock to maximise N_e_ and reduce domestication in the stocking program. Parentage analysis of wild‐caught fish is crucial for demonstrating natural recruitment (Steffensen et al. 2019). Because tissue or DNA samples are no longer available for some historical broodstock, microsatellites remain necessary for identifying certain stocked individuals. However, the increased resolution GTseq markers provides a valuable complementary tool for confirming the parentage of wild‐spawned fish. In cases where broodstock were not directly sampled, GTseq genotypes of unsampled parents may also be partially reconstructed using genotypes from known offspring that were previously validated with microsatellite data. Parentage analysis is also used to match wild‐caught age‐0 sturgeon captured downstream of telemetered adults involved in spawning behaviour to link habitat and environmental cues with successful sturgeon spawning (Bulliner et al. 2016).

## 5 Conclusions

Hybridisation poses a major threat to endangered species by eroding genetic integrity and increasing extinction risk. Although a conventional panel of 12–20 microsatellite markers can detect species and early‐generation hybrids, their resolution declines in backcrossing and introgression, where genetic differentiation become increasingly ambiguous. Over the last decade, conservation genetics has shifted toward SNP markers due to their abundance and ease of development. When coupled with GT‐seq approach, it provides high‐throughput, cost‐effective platform that has become widely adopted for monitoring species of conservation concern (Bootsma et al. 2020; Burgess et al. 2022; Campbell et al. 2015; Chang et al. 2021; Harris et al. 2024; Li et al. 2023; May et al. 2020; Meek and Larson 2019; Schmidt et al. 2020). Our newly developed GT‐seq panel with hundreds of loci that can be efficiently scored offers dual functionality for Pallid Sturgeon conservation. It provides high‐resolution identification of pure individuals versus hybrids (including backcrosses), and enables accurate parentage assignment to distinguish wild‐origin/hatchery‐origin fish and trace parentage of wild‐spawned offspring. These capabilities are beneficial for evaluating hatchery effectiveness and informing adaptive management. Beyond this application, GT‐seq panels have been proven effective in examining population structure (Arpin et al. 2024; Garrett et al. 2024; Hayward et al. 2022) and advancing to microhaplotype‐based tools for finer genetic resolution (Hargrove et al. 2024; Petrou et al. 2025). As such, the panel presented here provides a robust tool for the genetic monitoring and conservation of imperilled species.

## Author Contributions

Junman Huang, Aaron J. DeLonay, Richard Flamio Jr. and Edward J. Heist conceived the study. Junman Huang, Richard Flamio Jr., Nathan R. Campbell and Edward J. Heist participated in marker selection. Amy C. Buhman curated fish fin clips. Junman Huang and Amy C. Buhman performed the GT‐seq experiment. Aaron J. DeLonay collected samples and was critically involved in producing gynogenetic sturgeon necessary for the ddRAD study that made the development of the GTseq markers possible. Richard Flamio Jr. and Edward J. Heist did the ddRAD‐seq analysis. Junman Huang and Edward J. Heist did the GT‐seq analysis. Junman Huang, Richard Flamio Jr., Nathan R. Campbell and Edward J. Heist wrote the manuscript. All authors have edited and approved the final version of the manuscript.

## Funding

Funding for this work was provided by the U.S. Army Corp of Engineers Award W912HZ‐22‐2‐0006 and Platte River Recovery Implementation Program.

## Conflicts of Interest

The authors declare no conflicts of interest.

## Supporting information


**Figure S1:** Workflow for the development of the GT‐seq *Scaphirhynchus* SNP panel. Initial SNP markers were filtered based on tests for Hardy–Weinberg equilibrium and linkage equilibrium, followed by the selection of highly informative loci based on measures of genetic divergence (F_ST_), and nucleotide diversity. Markers with sufficient flanking sequences were used for GT‐seq multiplex PCR primer design. Primer sets were optimised through iterative testing for multiplex PCR performance, informativeness and genotype concordance. Loci exhibiting low amplification rates, high levels of primer pairs mismatches and excess heterozygosity were subsequently removed.
**Figure S2:** Sensitivity analysis of simulated parentage assignment in Cervus under varying minimum typed loci thresholds (left panels: A and C) across two marker panels (indicated by line colour), and proportions of sampled parents (right panels: B and D) testing in p‐loci panel. Analyses were conducted at two confidence levels: relaxed (80%, dot‐dashed lines) and strict (95%, solid lines). The top row (A, B) presents assignment rates, representing the proportion of offspring correctly assigned to their true parents. The bottom row (C, D) shows critical Delta values, indicating the confidence of parentage assignment.

**Table S1:** 120 sample information for *Scaphirhynchus* GT‐seq marker panel development.
**Table S2:** 428 baseline sample and genotype information for Pallid and Shovelnose Sturgeon used for Scaphirhynchus GT‐seq marker optimisation and validation.
**Table S3:** Genotype of 85 samples with shared ddRAD‐seq and GT‐seq data in 356 SNP loci for concordant analysis.
**Table S4:** 126 broodstock info and microsatellite genotype.
**Table S5:** Parameter settings of simulation of parentage analysis with sex known in Cervus.
**Table S6:** 267 probe sequences.
**Table S7:** 267 primer sequences.
**Table S8:** NewHybrids results of 428 baseline samples in 19 microsatellite panel.
**Table S9:** NewHybrids results of 428 baseline samples in 267 SNP panel.
**Table S10:** Allele frequency of Pallid Sturgeon for GT‐seq 267 SNP panel.
**Table S11:** Allele frequency of Shovelnose Sturgeon for GT‐seq 267 SNP panel.
**Table S12:** Allele frequency of Pallid Sturgeon for 19 microsatellite panel.
**Table S13:** Allele frequency of Shovelnose Sturgeon for 19 microsatellite panel.
**Table S14:** Genotype frequency of Pallid Sturgeon for GT‐seq 267 SNP panel.
**Table S15:** Genotype frequency of Shovelnose Sturgeon for GT‐seq 267 SNP panel.
**Table S16:** Genotype frequency of Pallid Sturgeon for 19 microsatellite panel.
**Table S17:** Genotype frequency of Shovelnose Sturgeon for 19 microsatellite panel.
**Table S18:** Heterozygosity for each locus in imputated genotype from P‐loci panel (126 broodstock).
**Table S19:** Heterozygosity for each locus in imputated genotype from S‐loci panel (126 broodstock).
**Table S20:** Heterozygosity for each locus in imputated genotype from SNP panel (126 broodstock).
**Table S21:** Heterozygosity for each locus in imputated genotype from usate panel (126 broodstock).

## Data Availability

Raw ddRAD‐seq reads are publicly available on Dryad (https://doi.org/10.5061/dryad.mcvdnck1x). Raw GT‐seq reads of baseline samples, along with genotype metrics, supplementary ddRAD‐seq processing documentation and program parameter settings are available on Dryad (https://doi.org/10.5061/dryad.m0cfxppj6). Custom scripts for simulation and plotting are available at https://github.com/JunmanHuang/rscript‐gtseq‐scaphirhynchus.
