# Supplementary material for: GT‐Seq Panel Development for Species Identification and Parentage Analysis of Closely Related Hybridising Scaphirhynchus Sturgeons
Source: Mol Ecol Resour. 2026 Mar 27;26(3):e70124. doi: 10.1111/1755-0998.70124 (PMC13031409; doi:10.1111/1755-0998.70124)
Supplement: Supplementary file 1 — Figure S1: Workflow for the development of the GT‐seq Scaphirhynchus SNP panel. Initial SNP markers were filtered based on tests for Hardy–Weinberg equilibrium and linkage equilibrium, followed by the selection of highly informative loci based on measures of genetic divergence (FST), and nucleotide diversity. Markers with sufficient flanking sequences were used for GT‐seq multiplex PCR primer design. Primer sets were optimised through iterative testing for multiplex PCR performance, informativeness and genotype concordance. Loci exhibiting low amplification rates, high levels of primer pairs mismatches and excess heterozygosity were subsequently removed. Figure S2: Sensitivity analysis of simulated parentage assignment in Cervus under varying minimum typed loci thresholds (left panels: A and C) across two marker panels (indicated by line colour), and proportions of sampled parents (right panels: B and D) testing in p‐loci panel. Analyses were conducted at two confidence levels: relaxed (80%, dot‐dashed lines) and strict (95%, solid lines). The top row (A, B) presents assignment rates, representing the proportion of offspring correctly assigned to their true parents. The bottom row (C, D) shows critical Delta values, indicating the confidence of parentage assignment. [file MEN-26-e70124-s002.docx]

This supplemental file corresponds to the paper titled “GT-seq panel development for species identification and parentage analysis of closely related hybridizing *Scaphirhynchus* sturgeons”.

Disclaimer: Any use of trade, firm, or product names is for descriptive purposes only and does not imply endorsement by the U.S. Government.

**Table of contents of figure legends:**

Fig. S1. Workflow for the development of the GT-seq *Scaphirhynchus* SNP panel. Initial SNP markers were filtered based on tests for Hardy-Weinberg equilibrium and linkage equilibrium, followed by the selection of highly informative loci based on measures of genetic divergence (F_ST_), and nucleotide diversity. Markers with sufficient flanking sequences were used for GT-seq multiplex PCR primer design. Primer sets were optimized through iterative testing for multiplex PCR performance, informativeness, and genotype concordance. Loci exhibiting low amplification rates, high levels of primer pairs mismatches and excess heterozygosity were subsequently removed.

Fig. S2. Sensitivity analysis of simulated parentage assignment in Cervus under varying minimum typed loci thresholds (left panels: A and C) across two marker panels (indicated by line color), and proportions of sampled parents (right panels: B and D) testing in p-loci panel. Analyses were conducted at two confidence levels: relaxed (80%, dot-dashed lines) and strict (95%, solid lines). The top row (A-B) presents assignment rates, representing the proportion of offspring correctly assigned to their true parents. The bottom row (C-D) shows critical Delta values, indicating the confidence of parentage assignment.


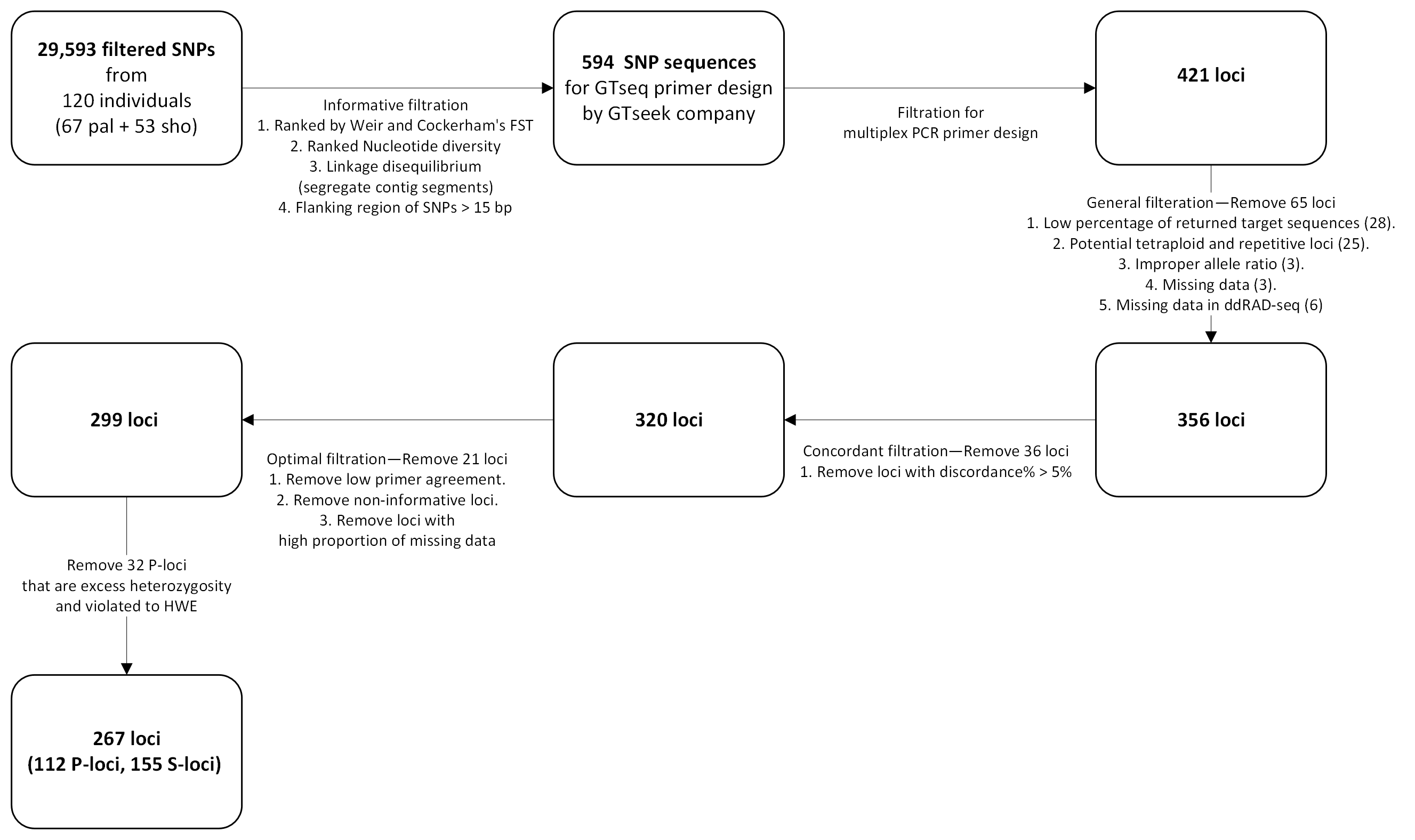
Fig. S1. Workflow for the development of the GT-seq *Scaphirhynchus* SNP panel. Initial SNP markers were filtered based on tests for Hardy-Weinberg equilibrium and linkage equilibrium, followed by the selection of highly informative loci based on measures of genetic divergence (F_ST_), and nucleotide diversity. Markers with sufficient flanking sequences were used for GT-seq multiplex PCR primer design. Primer sets were optimized through iterative testing for multiplex PCR performance, informativeness, and genotype concordance. Loci exhibiting low amplification rates, high levels of primer pairs mismatches and excess heterozygosity were subsequently removed.


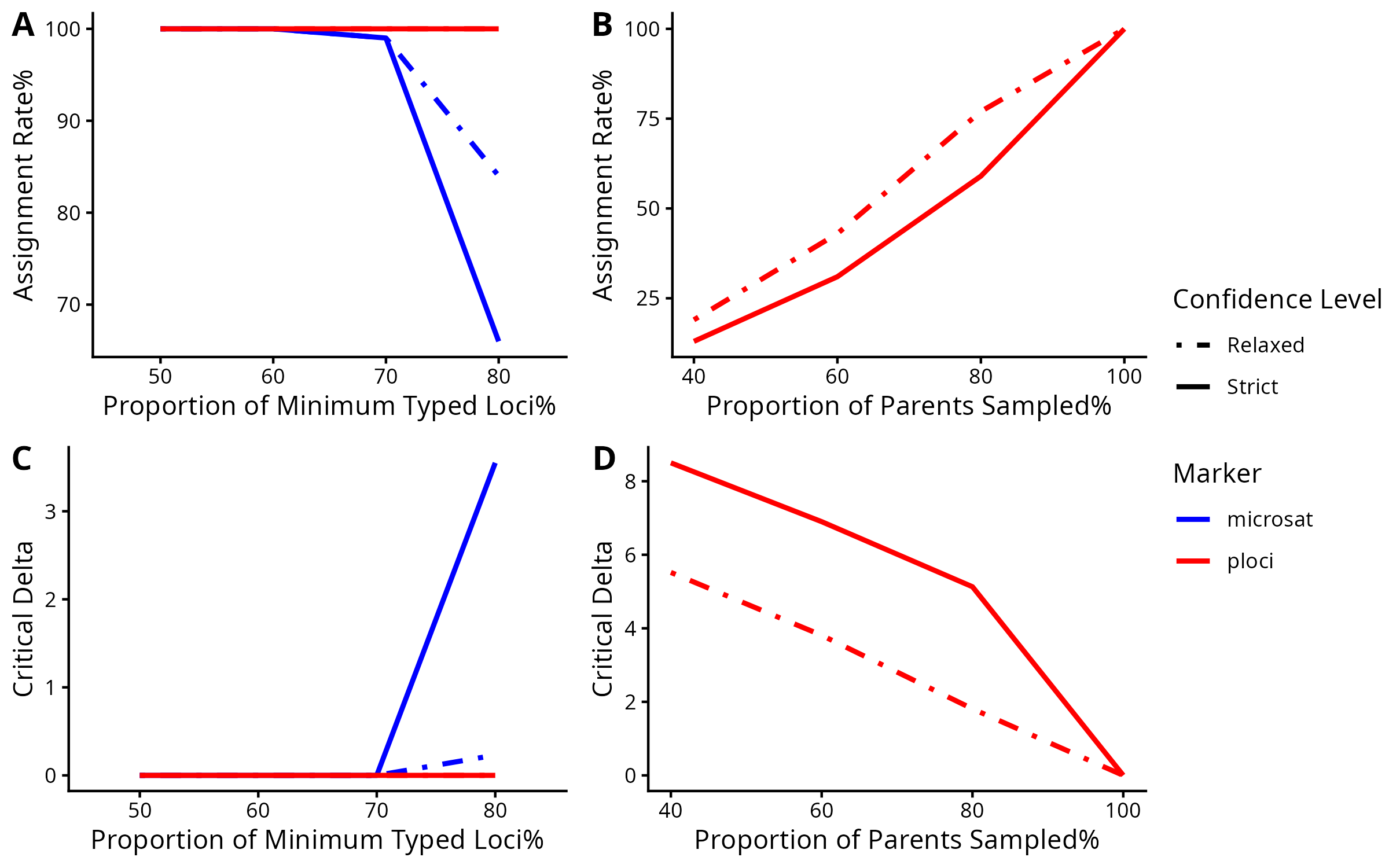
Fig. S2. Sensitivity analysis of simulated parentage assignment in Cervus (Kalinowski et al. 2007) under varying minimum typed loci thresholds (left panels: A and C) across two marker panels (indicated by line color), and proportions of sampled parents (right panels: B and D) testing in p-loci panel. Analyses were conducted at two confidence levels: relaxed (80%, dot-dashed lines) and strict (95%, solid lines). The top row (A-B) presents assignment rates, representing the proportion of offspring correctly assigned to their true parents. The bottom row (C-D) shows critical Delta values, indicating the confidence of parentage assignment.

**Reference**

Kalinowski, S. T., Taper, M. L., & Marshall, T. C. (2007). Revising How the Computer Program Cervus Accommodates Genotyping Error Increases Success in Paternity Assignment. *Molecular Ecology*, *16*(5), 1099–1106. <https://doi.org/10.1111/j.1365-294X.2007.03089.x>
